# Exploring working conditions and job stability of midwives in Spain: results from a cross-sectional national survey

**DOI:** 10.1186/s12884-026-09484-5

**Published:** 2026-06-17

**Authors:** Noelia Rodríguez-Blanco, Jesús Sánchez-Más, Diego Ayuso-Murillo, Mª Guadalupe Fontán-Vinagre, Montserrat Angulo-Perea, Adriana Díaz-Gautier, Roberto Guerrero-Menéndez

**Affiliations:** 1 Research Group for Quality of Life and Health Research, Department of Nursing, Faculty of Health Sciences, Universidad Europea de Valencia, Valencia, Spain; 2 Biomedical Sciences Department, Health Sciences Faculty, CEU-Cardenal Herrera University, Elche, Spain; 3 Consejo General de Colegios Oficiales de Enfermería de España, Madrid, Spain; 4 Fundación Instituto Español de Investigación Enfermera, Consejo General de Colegios Oficiales de Enfermería de España, Madrid, Spain

**Keywords:** Midwifery, Working conditions, Temporary workers, Continuous training, Clinical competencies

## Abstract

**Background:**

Midwives are essential to perinatal care in Spain’s healthcare system, with roles expanding due to demographic changes to encompass technology integration, multidisciplinary collaboration, and pharmacotherapeutic management. Challenges persist, including suboptimal remuneration, competency misalignment, and employment precarity (92.7% temporary contracts in 2024), aggravating professional dissatisfaction, reduced job satisfaction, and well-being deficits. Influential factors include workload, contractual stability, and work–life balance, emphasizing the imperative for empirical analysis of labour conditions. This study describes midwives’ perceptions of Spanish work environments and assesses contract-type variations.

**Methods:**

We conducted a cross-sectional, quantitative survey of registered midwives in Spain using a self-administered, 23-item, web questionnaire (five domains) on EUSurvey to assess working conditions, capturing demographics, job characteristics, training/competence, and entitlements, and evaluating differences in professional perceptions by contract type. Bivariate analyses and multivariable logistic regression models (adjusted for age group and years of professional experience) were performed to evaluate differences in professional perceptions by contract type.

**Results:**

Of the 2,499 responses, 2,310 met the requirements (27.8% of practicing midwives in Spain). The majority worked in public hospitals (66.1%); 43.4% had permanent contracts, a figure that increased with age and experience. Temporary shiftcts, more common in the private sector, were associated with lower salaries and less salary satisfaction (mean scores 4.0–4.1 vs. 4.6 on a 10-point scale; *p* < 0.001) and greater exposure unfavourable working conditions due to staff shortages (requests for off-hours shifts: 90.2%, denied leave: 55.4%, approved leave cancelled: 22.8%; *p* < 0.001). Role intrusion was frequent (77.1%). Despite widespread participation in continuous training, only 15.8% felt fully competent in all core competencies.

**Conclusions:**

Employment temporariness and staffing shortages are associated with poorer working conditions, greater role intrusion, and low pay satisfaction among midwives. Despite access to training, key competencies remain under-implemented, highlighting the need for greater job stability, adequate staffing, and equitable professional development.

**Supplementary Information:**

The online version contains supplementary material available at 10.1186/s12884-026-09484-5.

## Background

Global healthcare sustainability faces significant challenges, including the growing burden of chronic diseases and recurrent infectious disease outbreaks. A critical concern is the projected shortage of health professionals. According to the World Health Organization (WHO), an estimated global deficit of 4.5 million nurses and 310,000 midwives is expected by 2030 [1, 2]. More recent estimates, however, suggest that the shortage of midwives alone could range from 710,000 to 980,000 by the same period [3].

Midwives play an essential role in women’s sexual and reproductive health across the lifespan, providing care during pregnancy, childbirth, and the postnatal period for both mothers and newborns [4, 5]. The third global *State of the World’s Midwifery 2021* report (SoWMy 2021) highlights five key benefits of investing in midwifery: improved health outcomes, increased workforce and economic activity, positive socioeconomic impact of healthcare investments, inclusive employment growth for women, and enhanced economic stability [6]. In this context, political action has been urged across European governments to strengthen investment in midwifery staffing, training, and working conditions [7].

In Europe, the median ratio of midwives is 9.1 per 10,000 women aged 14–65, whereas Spain reports one of the lowest ratios at 6.1, thereby constraining workforce capacity and limiting the ability to provide equitable and safe coverage of care demands [4]. To qualify as a midwife in Spain, candidates must first obtain registration as a general nurse and then complete a two-year specialization in Obstetric-Gynaecologic Nursing, a programme known by its Spanish acronym EIR (*Enfermero Interno Residente*) [8]. This pathway differs from qualification models in other European countries, such as Germany and France, where candidates directly access a bachelor’s degree in Midwifery, or the United Kingdom, where both models coexist [9].

The Spanish core curriculum for midwives was established in 2009 and aligns with a practice framework encompassing care delivery both in primary and acute care settings across public and private sectors. In primary care, the role of the midwife focuses on health promotion, prevention, and education in sexual and reproductive health, as well as pregnancy follow-up, postpartum care, and community-based newborn care. In hospital settings, midwives deliver specialised care during pregnancy, childbirth, and the postpartum period, assist births, care for newborns, detect risks, and collaborate within multidisciplinary teams [10]. However, demographic and social transformations in Europe have generated new care demands, expanding midwives’ roles beyond traditional perinatal care [11]. These demands include adopting new technologies, collaborating effectively with other healthcare professionals, and managing the prescription, dispensation, and administration of medicines and health products [5].

Despite midwives’ recognition as key healthcare providers within the Spanish health system, unresolved issues remain regarding working conditions, including salary, rest breaks, job stability, and workload, as well as the alignment of professional competencies with evolving care needs [12, 13]. These limitations hinder professional development, contributing to lower job satisfaction and negatively impacting well-being among midwives [13, 14]. Multiple factors have been identified as influential to job satisfaction and well-being, including physical work environment, workload demands (e.g., shift work and overload), job description, financial and contractual conditions (e.g., salary adequacy and job stability), role performance, technological changes, interpersonal relationships, professional development opportunities, organizational characteristics, and work–family balance [15, 16].

In relation to interpersonal relationships and professional development, effective interprofessional collaboration is essential for woman-centred care, as it recognises distinct professional roles [17]. When collaboration is inadequate, role disputes may arise, potentially resulting in role blurring or role intrusion. Role intrusion occurs when professionals overstep their specific competencies, encroaching on others’ responsibilities [18] Although rarely reported, the sustainable expansion of scope of practice in healthcare professions underscores the need for clear policy and practice frameworks to ensure safe, effective care [19].

Job stability remains a major weakness in the Spanish healthcare system due to the high prevalence of temporary employment among health professionals. Spanish labour regulations define three types of contracts based on duration: permanent contracts, typically obtained through competitive selection processes involving examinations and curriculum assessment; interim contracts, which are temporary without a fixed term and used for substitutions or to cover positions pending permanent allocation or leave of absence; and temporary contracts, which have a defined duration based on production needs [20].

Following the 2022 amendment to Spanish healthcare workforce regulations, healthcare professionals may remain in interim positions for a maximum of three years [21]. If the position has not been filled permanently within this period, the interim professional is offered a non-permanent, undefined-term contract, which provides greater stability and protection. Prior to this modification, more than 40% of the Spanish healthcare workforce was employed under interim or temporary contracts, in some cases for notably long periods [22]. In 2024, temporary contracts accounted for 92.7% of all new contracts signed by midwives. Across all contract types—permanent, interim, and temporary—23.2% were part-time positions, and 94% of these were temporary [23].

Employment instability limits professional development and increases the risk of well-being problems [24]. It is often linked to job insecurity, limited access to training, and exposure to workplace discrimination, and may also be associated to adverse employment affecting certain professionals [25]. The existing literature on midwives’ working conditions remains limited among the sources reviewed. However, a comparable study was conducted with a smaller sample in the same geographical context, although it did not focus specifically on job stability [13].

The relationship between employment instability and working conditions, such as salary, workload, continuous training and unfavourable working conditions, remains unclear. This study therefore aims to describe midwives’ perceptions of working conditions in Spain, including the implementation of developed competencies, and to examine differences according to contract type.

## Materials and methods

### Study design

This study employed a cross‑sectional survey design using a self‑administered, web‑based questionnaire to assess the working conditions of registered midwives in Spain. The reporting of this study adheres to the Strengthening the Reporting of Observational Studies in Epidemiology (STROBE) statement [26] and follows the Consensus-Based Checklist for Reporting of Survey Studies (CROSS) [27].

### Questionnaire details

The tool was developed by the research team in collaboration with 15 expert midwives representing different Spanish regions (Autonomous Communities). The self-administered survey was uploaded to the EUSurvey platform (v1.5.3, Public License, European Commission). As the instrument primarily comprised nominal variables, formal psychometric validation was not applicable; instead, expert review and pilot testing were used to support content validity and clarity. Five members of the expert panel assessed the first draft of the questionnaire and adapted items accordingly. The initial version of the tool was piloted with a sample of 25 midwives to ensure proper functionality, improve ease of completion, and estimate the time required to complete the survey.

The final version of the questionnaire included 23 items grouped into five sections: demographic characteristics, work status, training, competencies, and unfavourable working conditions. The variables were classified as demographic characteristics (age group), job details (years of experience, type of contract, area of practice, involvement in research and teaching, annual income, care time per patient, role intrusion), and training and competencies (participation in continuous training, perception of implemented competencies, and competency gaps). The comparative variable was the type of contract, which was grouped into three categories: permanent, interim, and temporary. The complete survey tool is provided in Supplementary Table 1.

### Sample characteristics

According to the census available on December 31, 2023; there were 8,301 active (non-retired) registered midwives in Spain [28]. A geographical quota sampling strategy was applied to ensure representation across the 17 autonomous regions and 2 autonomous cities of Spain. The study included registered midwives actively working in the Spanish healthcare system, while non-practicing midwives, midwifery trainees, and retired midwives were excluded. Based on a 95% confidence level, a 0.05 margin of error, and an assumed proportion of 0.5, the required sample size was calculated as 387 respondents. Details of the quota distribution are depicted in Supplementary Fig. 1.

### Survey administration

The questionnaire was administered through an EUSurvey link. Based on the pre-test, the estimated completion time was approximately 10 min, although no time limit was imposed. All items in the questionnaire were mandatory, except for one conditional follow-up item (“Staff shortage is associated with work overload”), which was displayed only to participants who reported staff shortages in their unit. This item had minimal missing data (*n* = 5). To ensure data integrity and prevent duplicate or automated responses, the platform required CAPTCHA verification prior to submission and restricted each IP address to a single response. The survey link was distributed nationwide through corporate emails and professional social media channels of the General Council of Nurses and the 52 regional Nursing Colleges in Spain, reaching registered midwives. Social media dissemination was conducted through the official accounts of the Council and the Spanish Institute of Nursing Research, both restricted to registered nurses and midwives. Participation was voluntary, and completion and submission of the questionnaire implied informed consent. An introductory cover page explained the study purpose and assured anonymity and confidentiality, emphasizing the absence of right or wrong answers. The survey remained open for responses throughout March 2024.

Respondent-related and measurement-related sources of common method bias (CMB) were addressed through procedural measures, including survey design strategies (e.g., clear instructions, assurance of anonymity, and avoidance of ambiguous items) and methodological separation (e.g., use of varied item formats and distinct measures for in- dependent and dependent variables) [29].

### Statistical analysis

Analyses were conducted using IBM SPSS Statistics software, version 29.0 (SPSS Inc., Chicago, IL, USA). All variables were categorical and are presented as counts and percentages. Bivariate associations between contract type (permanent, interim, temporary) and the study outcomes were examined using contingency tables and Pearson’s Chi-square tests.

To assess the independent predictive strength of contract type while controlling for confounding, multivariable logistic regression models were performed. Contract type was included as the main independent variable (permanent contracts as reference category). Covariates were selected a priori based on theoretical relevance from the literature and empirical associations in the current dataset. Final models were adjusted for “age group” and “years of professional experience”, as these variables showed strong associations with contract type (Table 1) and several outcomes. Additional sensitivity analyses including workplace setting (public hospital, primary care) were conducted when relevant. Results are reported as adjusted odds ratios (OR) with 95% confidence intervals (95% CI). Model goodness-of-fit was evaluated with the Hosmer-Lemeshow test. A p-value < 0.05 was considered statistically significant.

**Table 1:** Distribution of participants’ demographics categorised by contract type

		Type of contract			
	Total	Permanent	Interim	Temporary	*p*-value
N	2310 (100)	1002 (43.4)	508 (22.0)	800 (34.6)	
Age group					
< 30 year	331 (14.3)	11 (3.3)	51 (15.4)	269 (81.3)	
31–50 year	1546 (66.9)	608 (39.3)	422 (27.3)	516 (33.4)	
51–60 year	289 (12.5)	250 (86.5)	27 (9.3)	12 (4.2)	< 0.001
61–67 year	144 (6.2)	133 (92.4)	8 (5.6)	3 (2.1)	
Work Experience					
< 2 years	135 (5.8)	3 (2.2)	10 (7.4)	122 (90.4)	
2–5 years	403 (17.4)	19 (4.7)	82 (20.3)	302 (74.9)	
6–10 years	537 (23.2)	93 (17.3)	199 (37.1)	245 (45.6)	< 0.001
11–20 years	720 (31.2)	418 (58.1)	180 (25.0)	122 (16.9)	
> 20 years	515 (22.3)	469 (91.1)	37 (7.2)	9 (1.7)	
Public hospital (Yes)	1527 (66.1)	629 (41.2)	291 (19.1)	607 (39.8)	< 0.001
Private hospital (Yes)	208 (9.0)	48 (23.1)	55 (26.4)	105 (50.5)	< 0.001
Primary care (Yes)	846 (36.6)	386 (45.6)	218 (25.8)	242 (28.6)	< 0.001
Private suite (Yes)	73 (3.2)	17 (23.3)	22 (30.1)	34 (46.6)	0.002
Home childbirth (Yes)	34 (1.5)	12 (35.3)	13 (38.2)	9 (26.5)	0.070
Research (Yes)	18 (0.8)	9 (50.0)	6 (33.3)	3 (16.7)	0.230
Teaching (Yes)	108 (4.7)	60 (55.6)	26 (24.1)	22 (20.4)	0.005

Data are presented as number of responses (percentage). *p-value* calculated using the Chi-square test according to contract type

## Results

A total of 2,499 responses were collected, with representation from all Spanish regions and a mean provincial participation rate of 36.9%. Participation rates by province are listed in Supplementary Tables 2 and represented in Supplementary Fig. 2. Of the total responses, 142 were excluded because they were completed by non‑practicing professionals and 47 were excluded because they were completed by midwifery trainees. Consequently, 2,310 responses from actively practicing registered midwives met the study inclusion criteria and were included in the analysis, representing 27.8% of the active population of registered midwives in Spain.

The distribution of sample demographics categorised by contract type is presented in Table 1. The most represented age group was 31–50 years. Most participants worked in direct patient care within public hospitals (66.1%), and 43.4% of respondents reported having permanent contracts.

The proportion of permanent contracts increased progressively with age and work experience, reaching up to 90% among midwives aged 61–67 years and those with more than 20 years of professional experience. Most permanent contracts were concentrated in public hospitals (41.2%), whereas temporary contracts were more prevalent in private healthcare settings (50.5%).

Table 2 presents the distribution of midwives’ responses regarding working conditions, continuous training, and competencies considered necessary within the healthcare system, categorised by contract type. Most midwives reported annual salaries between €35,000 and €45,000. In bivariate analysis, temporary contracts were associated with lower salaries and significantly lower salary satisfaction (mean 4.0 ± 2.2) compared with permanent contracts (4.6 ± 2.3; *p* < 0.001). This difference remained statistically significant after adjustment for age and years of experience in multivariable logistic regression (see Supplementary Table 3).

**Table 2:** Working conditions including continuous training and competencies categorised by contract type

	Total	Permanent	Interim	OR (95% CI)	*p*-value	temporary	OR (95% CI)	*p*-value
*N* (%)	2310 (100)	1002 (43.4)	508 (22.0)			800 (34.6)		
WORKING CONDITIONS								
Income (€/year)								
< 25,000	362 (15.7)	65 (18.0)	53 (14.6)	6.02 (3.04–11.9)	< 0.001	244 (67.4)	72.1 (28.2-184.5)	< 0.001
25,000–30,000	770 (33.3)	234 (30.4)	207 (26.9)	6.53 (3.55-12.0)	< 0.001	329 (42.7)	27.0 (10.8–67.4)	< 0.001
30,000–45,000	1064 (46.1)	607 (57.0)	235 (22.1)	2.86 (1.57–5.20)	< 0.001	222 (20.9)	7.02 (2.82–17.5)	< 0.001
> 45,000 (Ref)	114 (4.9)	96 (84.2)	13 (11.4)	-	-	5 (4.4)	-	-
Time per patient*								
Yes (Ref)	202 (23.9)	107 (53.0)	53 (26.2)	-	-	42 (20.8)	-	-
No, 5 min/patient	292 (34.5)	127 (43.5)	79 (27.1)	1.22 (0.80–1.87)	0.354	86 (29.5)	1.56 (1.02–2.42)	0.039
No, 10 min/patient	352 (41.6)	152 (43.2)	86 (24.4)	1.18 (0.79–1.78)	0.418	114 (32.4)	1.85 (1.23–2.79)	0.003
CONTINUOUS TRAINING/COMPETENCIES								
Continuous training								
No (Ref)	45 (1.9%)	25 (55.6)	9 (20.0)	-	-	11 (24.4)	-	-
Yes	2265 (98.1)	977 (43.1)	499 (22.0)	1.42 (0.66–3.06	0.373	789 (34.8)	1.84 (0.90–3.75)	0.096
Fully Implemented competencies								
No (Ref)	1944 (84.2)	809 (41.6)	424 (21.8)	-	-	711 (36.6)	-	-
Yes	366 (15.8)	193 (52.7)	84 (23.0)	0.83 (0.63–1.10)	0.196	89 (24.3)	0.53 (0.40–0.69)	< 0.001
Role intrusion witnessed								
No (Ref)	528 (22.9)	275 (52.1)	82 (15.5)	-	-	171 (32.4)	-	-
Yes	1782 (77.1)	727 (40.8)	426 (23.9)	1.97 (1.49–2.59)	< 0.001	629 (35.3)	1.39 (1.12–1.73)	0.003
Acted against role intrusion								
No (Ref)	2178 (94.3)	946 (43.4)	470 (21.6)	-	-	762 (35.0)	-	-
Yes	132 (5.7)	56 (42.4)	38 (28.8)	1.37 (0.89–2.09)	0.152	38 (28.8)	0.84 (0.55–1.29)	0.427

Data are presented as number of responses (percentage). *OR* = odds ratio, estimated using permanent contract type as the reference category; *p-value* = calculated using the Chi-square test. *For the item “The time assigned for direct care per patient is adequate,” only midwives practicing in Primary Care were included in the analysis (*n* = 846)

**Fig. 1:**
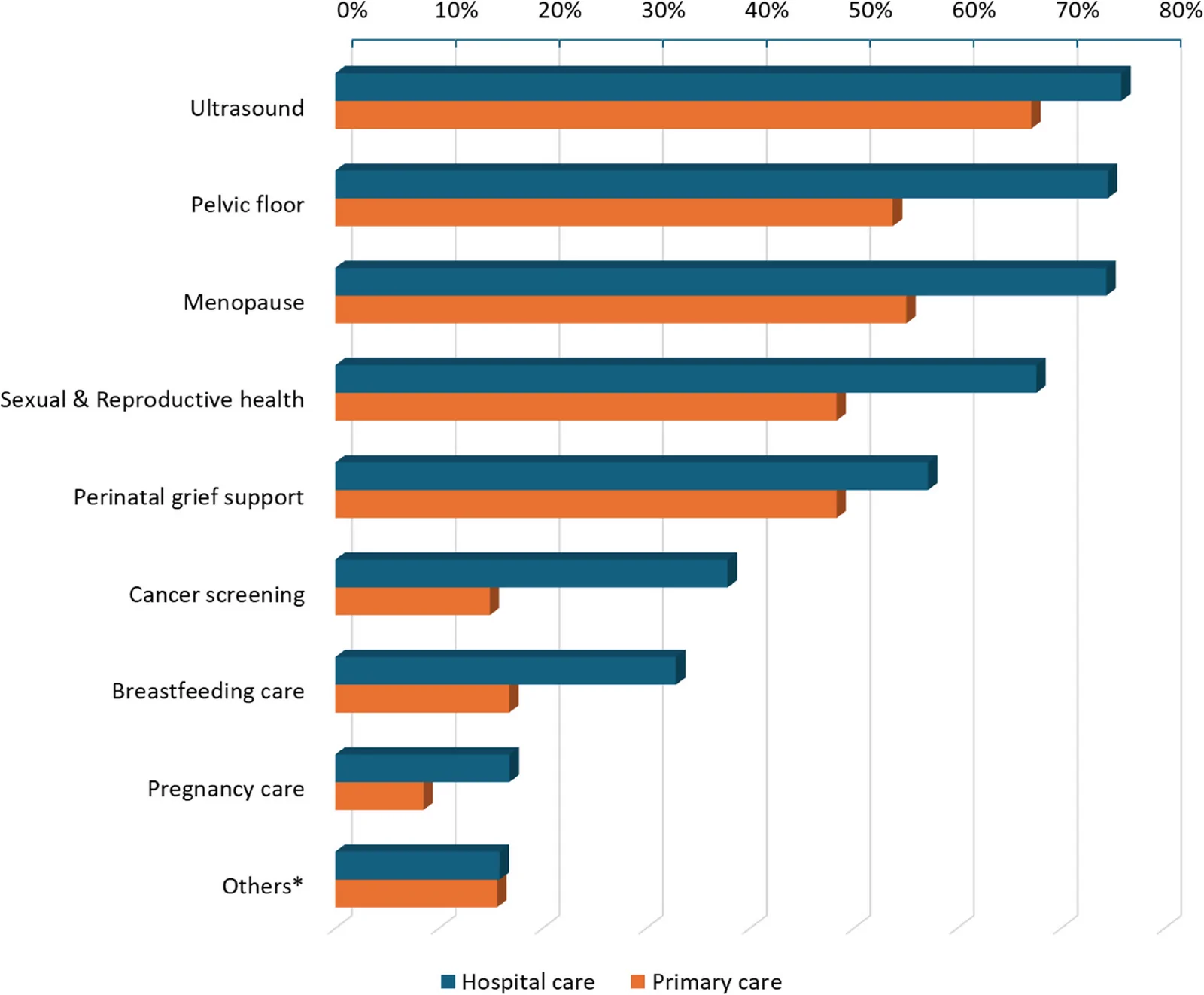
Midwives’ perceptions of implementation gaps across competencies in a multiple-choice item. Response rates among hospital care (*n* = 1,464) and primary care (*n* = 846) midwives * Other answers included in an open conditional item were home postnatal care; sexually transmitted infections (STIs) in adolescents; assisted reproduction and fertility; puberty and menstrual cycle care; perinatal mental health; teaching; and research.

Most midwives working in Primary Care (76.1%) considered it necessary to increase the time allocated for direct patient care, a concern significantly more pronounced among those with temporary contracts compared to permanent employees. Most participants (77.1%) reported witnessing role intrusion, although only 5.5% had formally taken legal actions. Nearly all respondents reported engagement in continuous training regardless of contract type; however, only 15.8% believed that all clinical competencies developed for safe midwifery practice are fully implemented in their region. The most frequently identified competency gaps included ultrasound, menopause care, sexual and reproductive health, perinatal grief care, and pelvic floor management, whereas pregnancy and breastfeeding care were most perceived as fully implemented (Fig. 1). Similar response patterns were observed between hospital and primary care midwives regarding competency gaps, although hospital-based midwives expressed greater concern about breastfeeding care and cancer screening competencies.

Table 3 summarises midwives’ unfavourable working conditions associated with the uncovered staff shortage in units, categorised by contract type. In bivariate analyses, midwives with temporary contracts were significantly more likely to experience requests for off-hours shifts (90.2% overall), denial of leave (55.4%), and cancellation of approved annual leave (22.8%) (all *p* < 0.001). These associations persisted after adjustment for age group and years of professional experience (OR for temporary vs. permanent contracts ranging from 2.1 to 3.8 across outcomes; all *p* < 0.001). Full adjusted models are presented in Supplementary Table 3. A total of 38.9% of participants reported limited availability of replacement staff to cover absences, including annual leave, part-time shifts, and sick leave. This lack of coverage was considered problematic by most respondents (98.3%), who believed it contributes to work overload.

**Table 3:** Unfavourable working conditions associated with midwife shortages, by contract type

	Total	Permanent	Interim	OR (95% CI)	*p*-value	Temporary	OR (95% CI)	*p*-value
*N* (%)	2310 (100)	1002 (43.4)	508 (22.0)			800 (34.6)		
Contracts to cover leave								
No (Ref)	898 (38.9)	386 (43.0)	235 (26.2)	-	-	277 (30.8)	-	-
Yes	1412 (61.1)	616 (43.6)	273 (19.3)	0.72 (0.59–0.90)	0.004	523 (37.0)	1.18 (0.98–1.44)	0.088
Staff shortage leads to work overload*								
No (Ref)	15 (1.7%)	11 (73.3)	2 (13.3)	-	-	2 (13.3)	-	-
Yes	876 (98.3)	371 (42.4)	233 (26.6)	3.45 (0.76–15.7)	0.109	272 (31.1)	4.03 (0.89–18.3)	0.071
Denied leave entitlements due to shortage of midwives								
No (Ref)	1031 (44.6)	492 (47.7)	242 (23.5)	-	-	297 (28.8)	-	-
Yes	1279 (55.4)	510 (39.9)	266 (20.8)	1.06 (0.86–1.31)	0.591	503 (39.3)	1.63 (1.35–1.98)	< 0.001
Cancelled annual leave due to staff shortages								
No (Ref)	1784 (77.2)	816 (45.7)	391 (21.9)	-	-	577 (32.3)	-	-
Yes	526 (22.8)	186 (35.4)	117 (22.2)	1.31 (1.01–1.70)	0.041	223 (42.4)	1.69 (1.35–2.12)	< 0.001
Cover extra shifts due to staff shortages								
No (Ref)	227 (9.8)	120 (52.9)	50 (22.0)	-	-	57 (25.1)	-	-
Yes	2083 (90.2)	882 (42.3)	458 (22.0)	1.25 (0.88–1.77)	0.216	743 (35.7)	1.77 (1.27–2.47))	< 0.001

Data are presented as number of responses (percentage). *OR* = odds ratio, estimated using permanent contract type as the reference category; *p-value* = calculated using the Chi-square test. *For the question “Staff shortage leads to work overload,” only 891 participants were included; the remaining respondents left this item blank

## Discussion

This study aimed to describe midwives’ perceptions of working conditions in Spain, focusing on the implementation of competencies, continuous training, and unfavourable working conditions, and examining how these are associated with contract type. The study found that job stability increased with age and years of experience. Midwives with temporary contracts had lower salaries and were less satisfied with their remuneration. They were also significantly more likely to experience unfavourable working conditions, including requests to work outside normal hours, denial of leave requests, and cancellation of approved annual leave. These associations remained significant after adjustment for age and professional experience, indicating that contract type exerts an independent effect beyond the confounding influence of younger age and lower experience.

The proportion of respondents working in the public sector was high but still lower than that reported in other studies conducted in comparable populations, such as previous research in Spain (95.6%) [13], the UK (88.6%) [30], and Australia (89.8%) [31]. Conversely, the proportion of midwives working exclusively in the private sector in Spain was similar to that observed in the UK (0.8%) and markedly lower than in Australia (10.6%).

Overall salary satisfaction among Spanish midwives was moderate to low, with particularly low levels reported by temporary and interim staff. Given that salary satisfaction is a known determinant of overall job satisfaction [32], this finding warrants further attention. Other studies, however, have highlighted additional components of job satisfaction beyond remuneration [33, 34].

Regarding time allocated for patient care, responses showed relatively little variability, with a slight preference for an additional 10 min per consultation. Similar concerns have been raised in international contexts. For instance, a study in Peru [35] questioned the quality of care delivered by primary care midwives due to the brevity of patient interactions, with midwives averaging 4–5 min per patient despite dedicating more total minutes per shift to direct care than nurses or physicians. Overall low satisfaction with workload can be deducted from our results, consistent with previous national results [13], while temporariness reveals significant differences. Nevertheless, further research is needed to explore the nature of midwives’ care activities in different healthcare settings to enable meaningful comparisons and to assess care needs from multiple perspectives.

### Training

In our study, most respondents obtained their midwifery qualification through the EIR pathway, a proportion consistent with the findings of Iglesias-Casas et al. [13], who reported that this training pathway graduates professionals who feel well-prepared, confident, and competent in their professional role. No differences were observed between contract types in relation to participation in continuous training, suggesting that engagement in continuous training was not age‑dependent. This contrasts with evidence from the nursing profession, where continuous training has been more commonly associated with older age groups in previous studies [36, 37].

### Competencies

According to the analysis reported by the Spanish Federation of Midwives’ Associations [3], Spanish midwives have one of the broadest ranges of competencies in Europe. Among the competencies included in midwives’ training programs, the most relevant are related to pregnancy care, childbirth, postnatal care, and women’s reproductive and sexual-affective health [10]. However, participants in our study reported that many of these competencies are not fully implemented in real practice. Most participants identified gaps in the implementation of developed competencies within their regional health services, while only midwives practicing in Catalonia and Navarre expressed some alignment between competencies acquired during training and those applied in clinical practice.

The major gaps were identified in ultrasound, menopause care, pelvic floor health, and reproductive and sexual health. This issue may be related to findings from a review conducted by Watkins et al. [38], which indicated that gaps in competency implementation are influenced by factors such as systemic fragmentation of services and negative perceptions of midwives within organizations, communities, and other professional disciplines. Further studies should explore geographical and social barriers to midwives’ competency implementation.

As a breach of professional recognition and development, midwives’ competencies may encounter interdisciplinary challenges, such as role intrusion [39]. Most respondents reported having witnessed role intrusion, although only a small proportion pursued legal action, with doulas being the most frequently cited professionals involved. While the proportion of midwives reporting role intrusion was slightly lower than that reported by Iglesias‑Casas et al. (92.4%) [13], this difference may be explained by our focus on witnessed cases rather than broader perceptions of its existence. Although role intrusion is scarcely addressed in the literature, it is perceived as a barrier to effective teamwork and may pose risks to both patients and providers [18, 39]. Our findings, together with the growing presence of doulas —who provide continuous physical, emotional, and informational support during the perinatal period— highlights the importance of clearly defined boundaries [40, 41]. In Spain, where the role of doulas is not formally regulated or integrated into the healthcare system, establishing clear practice limits between regulated healthcare professionals and other agents is essential to prevent role intrusion and promote safe, collaborative care [42].

### Unfavourable working conditions

Our data show that midwives with temporary contracts were more likely to experience unfavourable working conditions, including working extra hours, covering additional shifts due to staff shortages, denial of requests for days off, and cancellations of previously approved annual leave. Importantly, these associations persisted in multivariable models adjusted for age and years of experience, suggesting that employment precarity itself contributes to poorer working conditions independently of the demographic profile of temporary workers.

Our findings are consistent with those of a recent cross-sectional study conducted among nurses in Greece, which concluded that temporary employment is associated with lower perceived job security and well-being, as well as a higher prevalence of mental health problems, irrespective of age and gender, and without significant effects on work capacity at the time of the study [43]. Additionally, studies conducted in Ireland [44], Sweden [45], Denmark [46], and Norway [47] have identified associations between mental health problems, reduced work capacity, wand intentions to leave the profession. However, longitudinal research designs, such as cohort studies, may be better suited to capturing the long-term effects of temporary employment on occupational health and retention.

Fagerström et al. [48] concluded that optimal management of healthcare staff is essential to achieve acceptable standards of care, and Griffith et al. [49] affirmed that quality of care improves when staffing levels exceed the minimum required threshold. In relation to this, evidence shows an association between daily workload per nurse and patient safety incidents and mortality [48], which is three to five times higher when workloads remain at medium-high levels compared to low levels (below optimal thresholds) [50].

Further research should extend to different nursing specialties and other healthcare disciplines in diverse contexts. This would allow exploration of functional and structural barriers within healthcare systems, supporting the adaptation of staffing and professional profiles to current care demands.

### Study limitations

This study has several limitations related to potential sources of population bias arising from the sampling method employed. The voluntary, survey‑based design may have introduced self‑selection bias, as midwives experiencing poorer working conditions or employment precarity may have been more motivated to participate, potentially leading to an overestimation of unfavourable working conditions. In addition, non‑response bias cannot be excluded, as midwives with more stable employment conditions may have been less likely to respond.

The midwifery workforce in Spain is highly feminised. National census data and evidence from a comparable study indicate that women represent over 90% of practicing midwives. Given this demographic imbalance, meaningful gender-based comparisons were not methodologically feasible; therefore, gender was not included as a variable in the survey instrument.

The Spanish census of midwives and the sample from a similar study comprised 91.2% and 95.6% women, respectively, indicating a highly feminised professional population [13, 28]. Given this demographic distribution, meaningful gender‑based comparisons would not be methodologically reliable and gender was not included as a variable in the survey tool.

This study did not collect specific data on full-time versus part-time employment. As working hours in Spain are flexible, dynamic, and not determined solely by contract type, this limited our ability to assess their impact on job stability, salary satisfaction, and working conditions.

Although procedural measures were implemented to mitigate common method bias (CMB), statistical techniques for its assessment could not be applied because they typically require continuous or normally distributed variables [51]. As most questionnaire items were nominal and descriptive, conventional validation methods were not applicable. Consequently, conclusions regarding the strength of the observed relationships should be interpreted with caution.

## Conclusions

Our study revealed that job temporariness among midwives is associated with poorer working conditions and reduced employment rights, including extra shifts, denial of denied days off, and cancellations of approved leave. Most respondents work in the public healthcare sector and report low to moderate salary satisfaction despite having sufficient time to direct patient care. Midwives highlight to expand and implement clinical competencies aligned with Spain’s specialised midwifery training and continuous professional development. Addressing persistent staff shortages and employment precarity is essential to protect care quality, professional equity and workforce well-being.

## Supplementary Information

Supplementary table 1. Survey tool about midwives’ working conditions, training, competencies and unfavourable working conditions; Supplementary figure 1. Geographical distribution of quota sampling; Supplementary table 2. Geographical distribution of responses by province; Supplementary figure 2. Geographical representation of response rates by province. Supplementary table 3. Multivariable logistic regression models assessing the association between contract type and key outcomes, adjusted for age group and years of professional experience.

## Data Availability

The datasets generated and/or analysed during the current study are available in the “Encuesta a Matronas” repository in the EUSurvey platform, (https://ec.europa.eu/eusurvey/publication/MATROENCUESTA2024) .
