# Supplementary material for: Exploring working conditions and job stability of midwives in Spain: results from a cross-sectional national survey
Source: BMC Pregnancy Childbirth. 2026 Jun 17;26:954. doi: 10.1186/s12884-026-09484-5 (PMC13508315; doi:10.1186/s12884-026-09484-5)
Supplement: Supplementary file 1 [file 12884_2026_9484_MOESM1_ESM.zip › Supplementary Figure 2.docx]

Supplementary figure 2. Geographical representation of response rates by province.

Response rate is expressed as the percentage of survey respondents relative to the number of registered midwives in each province. * The data on active registered Specialist Nurses in Obstetrics and Gynaecology (midwives) were provided by the Official Colleges of Nurses in each province to the National Institute of Statistics of Spain (INE). [24].
