# Supplementary material for: Exploring working conditions and job stability of midwives in Spain: results from a cross-sectional national survey
Source: BMC Pregnancy Childbirth. 2026 Jun 17;26:954. doi: 10.1186/s12884-026-09484-5 (PMC13508315; doi:10.1186/s12884-026-09484-5)
Supplement: Supplementary file 1 [file 12884_2026_9484_MOESM1_ESM.zip › Supplementary table 1.docx]

**Supplementary table 1.** Survey tool about midwives’ working conditions, training, competencies and adverse employment actions.

**DEMOGRAPHICS**

1. **What province are you registered in?**  52 options

2. **Age**

- < 30 years

- 31-50

- 51-60

- 61-67

**WORKING CONDITIONS**

3. **Do you currently practice as a midwife?**^a^

- I am currently qualifying as midwife

- No

- Yes

4. **Reason for no practice^b^**

- I have a permanent position as a general nurse

- I have a stable nurse contract as a general nurse, and I am only offered short contracts as midwife

- I am unemployed

- I am on sick leave or leave of absence

- Others

5. **What area do you work in? (you can choose more than one option)**

- Public hospital

- Private hospital

- Primary Care

- Birthplace House

- Research

- Home Birth Care

- Teaching

- Private Consultation

6. **How long have you been practicing as midwife?**

- 1 year

- 2-5 years

- 6-10 years

- 11-20 years

- >20 years

7. **What type of contract do you currently have as a midwife?**

- Temporary contract

- Interim contract

- Permanent contract

8. **What is your gross annual income?**

- Less than or equal to €25,000

- 25.001-30.000€

- 30.001-45.000€

- > 45.001€

9. **Do you work paid overtime?**

- No

- Yes

10. **To what degree do you consider your work to be well paid?**

To be valued from "0" which corresponds to "nothing" and "10" which corresponds to "very good"

11. **Do you consider that the time frame you have assigned for the care of each person is adequate?** **^c^**

- Yes.

- No, I would need an average of 5 min. more per person

- No, I would need an average of 10 min. more per person

12. **Have you ever been asked to cover shifts outside your regular working hours due to staff shortages?**

- No

- Yes

**TRAINING**

13. **How did you qualify as midwife?**

- Via EIR programme

- Training as a midwife in another country

- Midwife by the route prior to the EIR programme

14. **Have you completed continuous training in the last two years?**

- No

- Yes

15. **What continuing education needs do you think should be added, due to its special interest for the profession?**

**COMPETENCIES**

16. **Do you consider that, in the regional healthcare system in which you work, all the skills of our profession are developed?**

- No

- Yes

17. **Which competencies do you think are missing? (you can choose more than one option)** **^d^**

- Menopause

- Sexual and reproductive health

- Breastfeeding Care

- Attention to perinatal grief

- Cancer screening programs

- Pelvic floor

- Pregnancy Care

- Ultrasound

- Other

18. **Do you know of cases of role intrusion?**

- No

- Yes

19. **Have you initiated legal actions against role intrusion?** **^e^**

- No

- Yes

**ADVERSE EMPLOYMENT ACTIONS**

20. **Does your service/centre hire midwives specifically to cover leave, holidays and reductions in working hours?**

- No

- Yes

21. **Do you think this could increase in the workload for care teams?** **^f^**

- No

- Yes

22. **Have you ever been denied a day leave for your own business or unpaid leave due to a lack of professionals to cover it?**

- No

- Yes

23. **On any occasion, after having authorized vacations, have you been forced to change them due to a lack of midwife professionals to cover the replacement?**

- No

- Yes

**a**. Items 4–23 are conditionally displayed only when the response to this question is “Yes.”; **b.** Item displayed only when the response to question 3 is “No.”; **c.** Item displayed only when the response to question 5 is “Primary care.”; **d.** Item displayed only when the response to question 16 is “No.”; **e.** Item displayed only when the response to question 18 is “Yes.”; **f.** Item displayed only when the response to question 20 is “No.”
