# Supplementary material for: Exploring working conditions and job stability of midwives in Spain: results from a cross-sectional national survey
Source: BMC Pregnancy Childbirth. 2026 Jun 17;26:954. doi: 10.1186/s12884-026-09484-5 (PMC13508315; doi:10.1186/s12884-026-09484-5)
Supplement: Supplementary file 1 [file 12884_2026_9484_MOESM1_ESM.zip › Supplementary Table 2.docx]

**Supplementary table 2.** Geographical distribution of responses by province.

| **Province** | **Active Registered midwives (N)*** | **Responses (n)** | **Rate (%)** |
| --- | --- | --- | --- |
| Alava | 77 | 35 | 45.5 |
| Albacete | 127 | 43 | 33.9 |
| Alicante | 311 | 113 | 36.3 |
| Asturias | 94 | 70 | 74.5 |
| Almería | 87 | 44 | 50.6 |
| Balearic Islands | 252 | 30 | 11.9 |
| Ávila | 34 | 21 | 61.8 |
| Badajoz | 85 | 12 | 14.1 |
| Cantabria | 122 | 41 | 33.6 |
| Barcelona | 736 | 139 | 18.9 |
| Burgos | 90 | 35 | 38.9 |
| Cáceres | 104 | 33 | 31.7 |
| Cádiz | 221 | 42 | 19.0 |
| Castellón | 66 | 18 | 27.3 |
| Ciudad Real | 85 | 18 | 21.2 |
| Madrid | 1.393 | 355 | 25.5 |
| Córdoba | 116 | 38 | 32.8 |
| Murcia | 280 | 49 | 17.5 |
| A Coruña | 246 | 48 | 19.5 |
| Navarre | 158 | 77 | 48.7 |
| Cuenca | 26 | 17 | 65.4 |
| Girona | 82 | 39 | 47.6 |
| La Rioja | 64 | 22 | 34.4 |
| Ceuta | 20 | 7 | 35.0 |
| Granada | 142 | 82 | 57.7 |
| Guadalajara | 33 | 25 | 75.8 |
| Melilla | 31 | 12 | 38.7 |
| Gipuzkoa | 200 | 40 | 20.0 |
| Huelva | 53 | 12 | 22.6 |
| Huesca | 51 | 14 | 27.5 |
| Jaén | 121 | 54 | 44.6 |
| León | 85 | 18 | 21.2 |
| Lleida | 63 | 34 | 54.0 |
| Lugo | 69 | 16 | 23.2 |
| Málaga | 322 | 35 | 10.9 |
| Ourense | 28 | 13 | 46.4 |
| Palencia | 35 | 10 | 28.6 |
| Las Palmas | 183 | 123 | 67.2 |
| Pontevedra | 146 | 39 | 26.7 |
| Salamanca | 78 | 21 | 26.9 |
| Santa Cruz de Tenerife | 202 | 96 | 47.5 |
| Segovia | 26 | 17 | 65.4 |
| Seville | 242 | 72 | 29.8 |
| Soria | 15 | 12 | 80.0 |
| Tarragona | 105 | 42 | 40.0 |
| Teruel | 22 | 8 | 36.4 |
| Toledo | 89 | 19 | 21.3 |
| Valencia | 420 | 101 | 24.0 |
| Valladolid | 104 | 18 | 17.3 |
| Biscay | 305 | 100 | 32.8 |
| Zamora | 42 | 16 | 38.1 |
| Zaragoza | 213 | 104 | 48.8 |
|  | **8.301** | **2499** | **36.9** |

* The data on active registered specialist Nurses in Obstetrics and Gynaecology (midwives) were provided by the Official Colleges of Nurses in each province to the National Institute of Statistics of Spain (INE). [24].
