# Supplementary material for: Exploring working conditions and job stability of midwives in Spain: results from a cross-sectional national survey
Source: BMC Pregnancy Childbirth. 2026 Jun 17;26:954. doi: 10.1186/s12884-026-09484-5 (PMC13508315; doi:10.1186/s12884-026-09484-5)
Supplement: Supplementary file 1 [file 12884_2026_9484_MOESM1_ESM.zip › Supplementary table 3.docx]

**Supplementary table 3.** Multivariable logistic regression models for the association between contract type and key outcomes, adjusted for age group and years of professional experience.

| **Outcome**  **(Dependent variable)** | **Contract type** | **aOR** | **95% CI** | **p-value** | **Nagelkerke R²** | **Hosmer-Lemeshow p** |
| --- | --- | --- | --- | --- | --- | --- |
| Salary satisfaction (Low vs. Medium-High) | Interim | 1.62 | 1.28-2.05 | <0.001 | 0.085 | >0.05 |
|  | Temporary | 1.89 | 1.52-2.35 | <0.001 |  |  |
| Time per patient (Yes) | Interim | 1.33 | 0.90-1.95 | 0.152 | 0.026 | >0.05 |
|  | Temporary | 0.84 | 0.53-1.34 | 0.463 |  |  |
| Continuous training (Yes) | Interim | 1.24 | 0.53-2.94 | 0.619 | 0.013 | >0.05 |
|  | Temporary | 1.54 | 0.58-4.08 | 0.389 |  |  |
| Fully Implemented competencies (Yes) | Interim | 0.79 | 0.58-1.09 | 0.151 | 0.019 | >0.05 |
|  | Temporary | 0.48 | 0.33-0.69 | <0.001 |  |  |
| Role intrusion witnessed (Yes) | Interim | 1.74 | 1.29-2.35 | <0.001 | 0.037 | >0.05 |
|  | Temporary | 1.19 | 0.88-1.62 | 0.260 |  |  |
| Acted against role intrusion (Yes) | Interim | 1.71 | 1.06-2.75 | 0.027 | 0.012 | >0.05 |
|  | Temporary | 1.28 | 0.73-2.24 | 0.388 |  |  |
| Contracts to cover leave (Yes) | Interim | 0.69 | 0.54-0.87 | 0.002 | 0.012 | >0.05 |
|  | Temporary | 1.08 | 0.83-1.40 | 0.580 |  |  |
| Staff shortage leads to work overload (Yes) | Interim | 1.50 | 1.18-1.91 | <0.001 | 0.012 | >0.05 |
|  | Temporary | 0.93 | 0.72-1.22 | 0.613 |  |  |
| Denied leave entitlements due to shortage of midwives (Yes) | Interim | 0.98 | 0.77-1.24 | 0.852 | 0.020 | >0.05 |
|  | Temporary | 1.44 | 1.11-1.87 | 0.006 |  |  |
| Cancelled annual leave due to staff shortages (Yes) | Interim | 1.35 | 1.01-1.80 | 0.040 | 0.018 | >0.05 |
|  | Temporary | 1.74 | 1.28-2.36 | <0.001 |  |  |
| Cover extra shifts due to staff shortages (Yes) | Interim | 1.04 | 0.70-1.54 | 0.847 | 0.035 | >0.05 |
|  | Temporary | 0.81 | 0.51-1.27 | 0.353 |  |  |

All models adjusted for age group and years of professional experience. Permanent contract is the reference category. aOR = adjusted Odds Ratio; 95% CI = 95% Confidence Interval. Hosmer-Lemeshow test p > 0.05 indicates adequate model fit in all cases.
